# ZooKeys 500: traditions and innovations hand-in-hand servicing our taxonomic community

**DOI:** 10.3897/zookeys.500.9844

**Published:** 2015-04-27

**Authors:** Terry Erwin, Pavel Stoev, Teodor Georgiev, Lyubomir Penev

**Affiliations:** 1 Hyper-diversity Group, Department of Entomology, MRC-187, National Museum of Natural History, Smithsonian Institution, Washington, P.O. Box 37012, DC 20013-7012, USA; 2 Pensoft Publishers, Sofia, Bulgaria; 3 National Museum of Natural History, Sofia, Bulgaria; 4 Institute of Biodiversity and Ecosystem Research, Bulgarian Academy of Sciences, Sofia, Bulgaria

On 27th of April 2015 ZooKeys published its jubilee issue 500. It has been exactly 28 months since we published our semiquincentennial issue (Penev et al. 2012) and made a review of the journal’s progress since its establishment in 2008. Now, reaching this milestone makes us cast a look back to see what we have achieved in the passed two and ⅓ years.

## And…we have a lot to be proud of !

**Figure 1.**
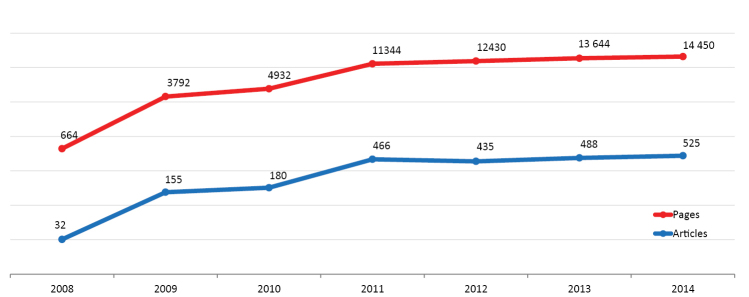
Total number of published articles and pages for the period 2008–2014.

From its start in July 2008 through April 2015, the journal published altogether 2436 articles and 65942 pages. The number of published articles continued to grow gradually over the last two years (see Fig. 1) reaching respectively 488 in 2013 and 525 in 2014. Likewise, the number of published pages increased from 12430 in 2012 to 14450 in 2014. The total number of submissions since the launch of the journal on 4^th^ of July 2008 reached 3407 or approximately 42 manuscripts per month, on average. Launched as a fast-line publishing journal, in spite of the great increase in submissions, the average peer-review and production time remained within the timeframes of 2012, namely approximately 3 months from submission to publication. The actual rejection rate based on evaluation of all submitted versus published articles is 28%.

Over the last two years, ZooKeys continued to increase its role in taxonomy sustained by implementing new publication models and technologies. In a race with the rapid destruction of ecosystems on the planet, the journal is seen as the best venue for describing the world’s biodiversity at a fast pace. Since 2008 until now, ZooKeys published altogether **5973 new taxa**, of which **5565 new species or subspecies**, **382 genera** and **26 families** (see also Table 1). It made its way to the top 10 journals publishing the greatest number of new taxa in Zoology reaching currently a second place in Thomson Reuters’ Index of Organism names right after Zootaxa. This accounts for 5.55% of all newly described animal taxa. In terms of nomenclature proposals it also ranks second with a share of 6.15% of all published acts (according to Thomson Reuters’ ION, accessed 18 April 2015). The journal is also in Zoological Record’s top ten publications containing new taxa with the publications of Marsh et al. (2013) and Fernández-Triana et al. (2014) on braconid wasps from Costa Rica, ranked respectively second and ninth.

**Table 1.** New taxa published in ZooKeys compared to all described animal taxa (data from Index of Organism Names, ZooBank and Pensoft’s Journal System).

Categories	Total described (all journals) 2009–2014 (ION)	Described in ZooKeys 2009–2014	% described in ZooKeys from all described 2009–2014
Species-group names	95377	5541	5.81%
Genus-group names	10204	381	3.73%
Family-group names	1501	26	1.73%
**Total**	**107082**	**5948**	**5.55%**

A number of technological and administrative measures were undertaken in the last two years to advance even more our journal’s position in the global publishing market. These were not left unnoticed by the scholarly community, data registries and leading indexers of scholarly literature. The ZooKeys impact factor, as evaluated by Thomson Reuters’ Journal Citation Reports for 2014, continue to grow and from 0.514 in 2010 reached 0.917 in 2013. Likewise, SCOPUS SJR indicator shows increase from 0.26 in 2010 to 0.48 in 2013.

In February 2013, Pensoft announced the integration of all its journals with CLOCKSS [Controlled LOCKSS (for Lots of Copies Keep Stuff Safe)] archive, which guaranteed their long-term preservation, integrity and perpetuity. As an added benefit, Pensoft became a partner of the Global LOCKSS Network supporting libraries and their local collections.

Being the first taxonomic journal to promote and implement data publishing in its routine workflow (Chavan and Penev 2011), over the last two years ZooKeys strengthened its leading position in this field and was recognised as the most reliable venue for publication, integration and dissemination of taxonomic data. From November 2011 when the first data paper was published in the journal (Narwade et al. 2011) until now, their number increased to 37 covering various aspects of biodiversity knowledge.

A major step towards strengthening the journal position was undertaken in December 2014 when ZooKeys moved on to a new technologically advanced publishing platform with several innovative features that better visualise published content and maximizes its re-use by readers. These include a navigation panel that allows key text elements, such as figures and tables to be downloaded individually. Other key features include visualisation of occurrence data on interactive Google map, Taxon Name Profile, and Reference finder (http://refindit.org). Besides, a new article level metrics allowing scoring the number of user’s visits by article format, as well as the number of views of each individual figure and table was introduced.

In November 2013, with the publication of issue 346 ZooKeys initiated an automated registration of new taxa with ZooBank. This was achieved through a server-to-server communication from the journal to ZooBank and back, using the TaxPub schema, which is an extension to the Journal Tag Publishing Suite (JATS) of the National Library of Medicine (NLM) (Catapano 2010, Penev et al. 2011). By doing this, ZooKeys became the first journal ever to implement such work flow in its publishing system and one of the first taxonomic journals accepted for archiving in PubMedCentral. Next to come is pipelining registration and publication of other types of nomenclatural acts.

The last two years will also be recalled with the publication of several landmark thematic monographs and conference proceedings, just to mention a few: Contributions to the systematics of New World macro-moths IV (Schmidt and Lafontaine 2013 – ZooKeys 264) and V (Schmidt and Lafontaine 2013 – ZooKeys 421); Advances in Hemipterology (Popov et al. 2013 – ZooKeys 319); DNA barcoding: a practical tool for fundamental and applied biodiversity research (Nagy et al. 2013 – ZooKeys 365); Review of taxonomy, geographic distribution, and paleoenvironments of Azhdarchidae (Pterosauria) (Averianov 2014 – ZooKeys 432); Proceedings of the Summer Meeting of the Crustacean Society and the Latin American Association of Carcinology, Costa Rica (Wehrtmann and Bauer 2014 – ZooKeys 457); The origin and early evolution of metatherian mammals: the Cretaceous record (Williamson et al. 2014 – ZooKeys 465).

Quite a number of interesting zoological discoveries were announced in the journal and attracted large audiences and considerable media interest (see also Tables 2 and 3). Among those, worth mentioning: a new procyonid mammal, the Olinguito, from the Andes (Helgen et al. 2013); a new genus of monk seals from the Caribbean Sea (Scheel et al. 2014); a new genus and several new species of bats from Africa and the Neotropics (Reeder et al. 2013; Velazco and Patterson 2014); a new subgenus and four new species of electric fishes from the Amazon and Congo river basins (Sullivan et al. 2013; Lavoué and Sullivan 2014); a new genus and species of rove beetles collected by Charles Darwin 180 years ago and published on his birthday (Chatzimanolis 2014); a new genus and species of ancient clams found in the depths of the Arctic Ocean (Valentich-Scott et al. 2014) and many others.

**Table 2.** Top 10 most accessed press releases of ZooKeys articles posted through EurekAlert! (from the EurekAlert! counter) for the period 1 December 2011–13 April 2015. The counter registers only downloads from EurekAlert! mostly by science media and journalists. The actual number of readers is actually much higher than this number.

Title	Author/s and year of publication of the original article	Date posted	Press release views on EurekAlert! website
*Megalara garuda*: the King of Wasps: A new, giant wasp comes from Indonesia	Kimsey and Ohl 2012	23-Mar-2012	44 669
World’s smallest frogs discovered in New Guinea	Kraus 2011	12-Dec-2011	44 247
Spider version of Bigfoot emerges from caves in the Pacific Northwest	Griswold et al. 2012	17-Aug-2012	16 361
Your small-living-creature shoots may benefit big science	Goula et al. 2013	30-Jul-2013	12 640
A new trout species described from the Alakir Stream in Antalya, Turkey	Turan et al. 2014	12-Dec-2014	9 602
Striped like a badger – new genus of bat identified in South Sudan	Reeder et al. 2013	9-Apr-2013	8 402
New scorpion discovery near metropolitan Tucson, Arizona	Ayrey and Webber 2013	19-Feb-2013	6 631
A new species of moth from the Appalachian Mountains named to honor the Cherokee Nation	Quinter and Sullivan 2014	25-Jun-2014/	6 466
Ninety-eight new beetle species discovered in Indonesia	Riedel et al. 2014	16-Dec-2014/	6 400
Mummy-making wasps discovered in Ecuador	Shimbori and Shaw 2014	8-May-2014	5 423

**Table 3.** Top ten most viewed articles of ZooKeys by unique views (according to the ZooKeys website counter accessed on 21 April 2015).

**Article**	**Views**
Helgen et al. 2013 – Taxonomic revision of the olingos (*Bassaricyon*), with description of a new species, the Olinguito	49 471
Griswold et al. 2012 – An extraordinary new family of spiders from caves in the Pacific Northwest (Araneae, Trogloraptoridae, new family)	48 181
Bouchard et al. 2011 – Family-Group names in Coleoptera (Insecta)	27 435
Hagedorn et al. 2012 – Creative Commons licenses and the non-commercial condition: Implications for the re-use of biodiversity information	24 237
Sereno 2012 – Taxonomy, morphology, masticatory function and phylogeny of heterodontosaurid dinosaurs	23 846
Winterton et al. 2012 – A charismatic new species of green lacewing discovered in Malaysia (Neuroptera, Chrysopidae): the confluence of citizen scientist, online image database and cybertaxonomy	22 629
Kraus 2011 – At the lower size limit for tetrapods, two new species of the miniaturized frog genus *Paedophryne* (Anura, Microhylidae)	17 054
Penev et al. 2009 – Data publication and dissemination of interactive keys under the open access model	16 988
Sereno and Larsson 2009 – Cretaceous Crocodyliforms from the Sahara	16 966
Cerretti et al. 2013 – A neotype designation for the bone-skipper Centrophlebomyia anthropophaga (Diptera, Piophilidae, Thyreophorina), with a review of the Palaearctic species of Centrophlebomyia	16 938
ICZN 2012 – Amendment of Articles 8, 9, 10, 21 and 78 of the International Code of Zoological Nomenclature to expand and refine methods of publication	15 578
**Total**	**279 323**

Shortly after the erection of the method of rapid and *en masse* descriptions of new taxa, often called “turbo-taxonomy” in 2012, ZooKeys served as an experimental testbed for the concept (Riedel et al. 2013, 2014). Furthermore, entirely new methodological approaches in taxonomy were introduced in the journal, among others a new LEGO pinned insect manipulator (IMp) (Dupont et al. 2015); a new illustration technique allowing integration of scanning electron microscope images into an interactive rotatable model (rSEM) (Akkari et al. 2013); a new set-up for production of highly detailed quality pictures of pinned insects (Brecko et al. 2014).

The journal success wouldn’t be possible without the great support of the zoological community. We deeply appreciate the help received from our most active authors, reviewers and editors!
